# Effects of a mat pilates program on cardiometabolic parameters in elderly women

**DOI:** 10.12669/pjms.292.3099

**Published:** 2013-04

**Authors:** Fourie Marinda, Gildenhuys Magda, Shaw Ina, Shaw Brandon, Toriola Abel, Daniel Ter Goon

**Affiliations:** 1 Fourie Marinda, Department of Sport, Rehabilitation and Dental Sciences, Tshwane University of Technology, Private Bag X680, Pretoria, Gauteng, 0001, Republic of South Africa.; 2 Gildenhuys Magda, Department of Sport, Rehabilitation and Dental Sciences, Tshwane University of Technology, Private Bag X680, Pretoria, Gauteng, 0001, Republic of South Africa.; 3 Shaw Ina, Office of the Deputy Pro Vice-Chancellor: Research, Monash South Africa, P.O. Box X60, Ruimsig, 1725, Republic of South Africa.; 4 Shaw Brandon, Department of Sport, Rehabilitation and Dental Sciences, Tshwane University of Technology, Private Bag X680, Pretoria, Gauteng, 0001, Republic of South Africa.; 5 Toriola Abel, Department of Sport, Rehabilitation and Dental Sciences, Tshwane University of Technology, Private Bag X680, Pretoria, Gauteng, 0001, Republic of South Africa.; 6 Daniel Ter Goon, Centre for Biokinetics, Recreation and Sports Science, University of Venda, X5050, Thohoyandou, Limpopo, 0590, South Africa.

**Keywords:** Pilates program, Resting heart rate, Resting blood pressure, Fasting blood glucose, Cholesterol, Triglycerides, Elderly women

## Abstract

***Objective:*** This study aimed to determine the effects of mat Pilates on resting heart rate, resting blood pressure and fasting blood glucose, cholesterol and triglycerides in elderly women.

***Methodology:*** Fifty sedentary, apparently healthy females aged 60 and older were randomly assigned into a control (CG, n = 25) or an intervention (IG, n = 25) group. The IG took part in an eight-week progressive mat Pilates exercise program, three times weekly while the CG did not take part in any structured exercises throughout the eight-week period. All subjects underwent pre- and post-tests in which cardiometabolic parameters were assessed.

***Results:*** In the eight-week mat Pilates program, the IG only demonstrated a significant (p ≤ 0.05) decrease in systolic BP (p = 0.040) from 135.84 ± 14.66mmHg to 128.80 ± 16.36mmHg and a significant increase in blood glucose (p = 0.000) from 5.07 ± 0.46mmol.L^-1^ to 5.83 ± 0.57mmol.L^-1^, whereas resting HR (p = 0.148) (from 68.80 ± 12.58beats.min^-1^ to 73.20 ± 11.46beats.min^-1^), resting diastolic BP (p = 0.342) (from 75.64 ± 10.10mmHg to 77.44 ± 9.32mmHg), blood TC (p = 0.073) (from 5.37 ± 0.99mmol.L^-1^ to 5.67 ± 1.04mmol.L^-1^) and blood TG (p = 0.384) (from 1.77 ± 0.88mmol.L^-1^ to 1.92 ± 0.87mmol.L^-1^) did not produce any significant changes.

***Conclusion:*** Due to the contradictory nature of the cardiometabolic variables (except systolic BP) with the findings of previous studies, it is difficult to establish a case for using Pilates as a substitute for more conventional forms of exercising when exclusively attempting to favourably alter cardiometabolic parameters at least among the elderly women in our sample.

## INTRODUCTION

With ageing maximum HR decreases^1^ due to the natural pacemaker (the sinoarterial (SA) node) losing some of its cells which may result in cardiac arrhythmias in the elderly, as well as incompetency among the valves inside the heart, which control the direction of blood flow.^2^ Ageing also results in a reduced elasticity in the arteries and possible reductions in left ventricular contractility.^1^ Another age-related finding was that with ageing, a decrease occurs in both the sympathetic and parasympathetic regulation of the heart^3^^-^^5^ and a reduced sensitivity of the heart to the body’s chemical catecholamine stimulation.^1^ The aorta also becomes thicker, stiffer and less flexible, which then results in an increase in BPblood pressure and causes the heart to work harder, which may lead to myocardial hypertrophy.^2^ However, this gradual stiffening and inability of the arteries to dilate and constrict, can explain the increases found in BP with ageing.^2^^,^^6^ Ageing also appears to be associated with specific and selective impairments in baroreflex function, which includes a decreased ability to alter cardiac period in response to acute alterations in BP and a decreased ability of the baroreflexes to buffer changes in systemic BP.^7^ Kidneys of the elderly also secrete less rennin,^2^ resulting in a decreased ability to respond to decreases in BP by eventually causing vasoconstriction and thus, BP to increase.

Ageing can also be seen as one of the largest factors affecting glucose homeostasis. According to Elahi and Muller and Stout,^8^^,^^9^ several glucose age-related responses occur, such as a decrease in glucose tolerance, inappropriate insulin secretion and insulin insensitivity occurs, where in turn, a number of possible mechanisms for glucose intolerance in the elderly exist. Glucose intolerance can be caused by changes in hormones involved in the increase uptake and suppression of glucose usage such as glucagon, growth hormone and pancreatic hormones.^9^ With ageing, there is also a tendency to increased total body weight and increased distribution of adipose tissue in the abdominal area, which mainly influence glucose tolerance by causing insulin resistance.^8^^,^^9^

With regards to lipids, Gostynski et al.^10^ found that the prevalence of dyslipidemia increases with age, however, there has been much debate whether cholesterol increases are the result of a natural process of intrinsic ageing or whether it is due to age-associated anthropometrics and/or lifestyle changes.^10^ Schubert et al.^11^ found that age is an important independent predictor for LDL-C and TC in men and TC in women, but it is not as influential as body composition and lifestyle on HDL-C and TG in men and women and LDL-C in women. Regardless of the link between overweight and cholesterol levels Nakagawa et al,^12^ found that intramyocellular lipid content also increases with age in lean elderly. This might be related to blood lipid and lipoprotein profiles, with a decrease in HDL-C and might affect health risks and muscle attenuation with age in lean elderly.

The weakening of the cardiovascular system associated with ageing, could be countered by increasing levels of physical activity and functional fitness.^13^ Pilates results in various physical improvements such as, increases in flexibility, bone density and dynamic balance as well as favourable changes in body mass index and have a positive effect on muscular strength and endurance.^14^^-^^17^ Correct breathing during Pilates can also prevent excessive stress on the heart, result in total body relaxation and mental calmness.^16^ This reduction in stress as a result of Pilates can reduce other serious complications. It may directly or indirectly result in an improvement in cardiometabolic parameters, especially in the elderly, resulting in reduced risk of developing coronary artery disease (CAD).^18^ However, little literature is available on the effects of Pilates on cardiometabilic parameters, therefore creating a need for scientific evidence on the beneficial effects of Pilates on cardiometabolic parameters.

## METHODOLOGY

A sample of 50 elderly female subjects (≥ 60 years of age), selected from caring facilities within Pretoria, Gauteng Province, South Africa were randomly assigned into one of two groups using a random numbers table; with 25 subjects undergoing an eight-week mat Pilates program (IG), while the other 25 subjects participated as a non-exercising control group (CG). The research protocol was approved by the Institutional Review Boards of the Tshwane University of Technology, Pretoria, South Africa and was endorsed by the International Physical Activity Projects (IPAP). Permission to conduct the study at the caring facilities was obtained from the relevant care facilities and all subjects signed a written informed consent form indicating all the advantages and risks involved of participation in the study. All subjects were required to obtain medical clearance prior to commencement of pre-testing procedures. Both groups took part in identical pre- and post-tests. Subject demographics at baseline are shown in Table-I.

All subjects were required to undergo cardiometabolic testing prior to and at completion of the eight-week treatment period. Each subject’s resting heart rate was measured in a seated position (after five minutes rest) by means of manually applying mild pressure. Pressure was applied to the radial artery on the lateral side of the anterior forearm just proximal to the wrist.^2^ Resting BP was measured by means of a sphygomanometer and a stethoscope (Jiangsu Dengguan Medical Treatment Instrument Co., Ltd.). The subject was required to be supine for five minutes and the tested arm was supported and the cuff was wrapped securely around the right arm at heart level. The stethoscope was placed below the cuff and the cuff inflated to approximately 180 millimeters mercury (mmHg). The pressure was then slowly released and different heart sounds were heard. The first sound heard was used as the systolic blood pressure (heart contraction) value and the sound heard when the tone changed or disappeared was used for the diastolic blood pressure (heart relaxation) reading.^2^ Fasting blood glucose, total cholesterol (TC) and triglycerides (TG) were measured by means of using the Reflotron system **(**F. Hoffmann-La Roche, Grenzarcherstrausse 124, CH-4070 Basel, Switzerland) after an eight-hour fast. A multiclix lancing device was used to prick the subjects’ finger. A drop of blood was then placed on a Reflotron testing strip and the test strip placed in the Reflotron. The glucose, cholesterol and triglycerides readings were then digitally displayed.

**Table-I:** Subject demographic data

	*Non-exercising control group (CG) * *n = 25*	*Mat Pilates program group (IG)* *n = 25*
Age (years)	65.32 ± 5.01	66.12 ± 4.77
Weight (kg)	75.19 ± 14.78	71.71 ± 14.92
BMI (kg.m^-2^)	29.32 ± 5.44	28.32 ± 6.77

Values are means ± standard deviation ± SD; kilograms per square meter

**Table-II:** Pre- and post-test cardiometabolic changes in the mat Pilates and non-exercising control groups

*Variables*	*Non-exercising control group (CG)* *n = 25*			*Mat Pilates program group (IG)* *n = 25*		
	*Pre-test*	*Post-test*	*p-value*	*Pre-test*	*Post-test*	*p-value*
Resting heart rate (beats.min^-1^)	62.48±8.98	74.92 ± 9.96	0.000*	68.80±12.58	73.20±11.46	0.148
Resting systolic blood pressure (mmHg)	134.48±13.65	136.00±17.83	0.705	135.84±14.66	128.80±16.36	0.040*
Resting diastolic blood pressure (mmHg)	81.36 ± 9.27	79.76 ± 9.31	0.471	75.64 ±10.10	77.44 ± 9.32	0.342
Glucose (mmol.L^-1^)	5.24 ± 0.71	5.75 ± 0.59	0.001*	5.07 ± 0.46	5.83 ± 0.57	0.000*
Total cholesterol (mmol.L^-1^)	5.27 ± 1.27	5.43 ± 1.27	0.054	5.37 ± 0.99	5.67 ± 1.04	0.073
Triglycerides (mmol.L^-1^)	1.62 ± 0.54	1.78 ± 0.71	0.321	1.77 ± 0.88	1.92 ± 0.87	0.384

Values are means ± standard deviation ± SD;

*: Indicates significant difference from pre- to post-test (p ≤0.05)

Mat Pilates exercises were derived from Worth^19^ in order to compile the mat Pilates exercise program. A session was allocated to explain the basics of mat Pilates, before commencement of the program. This session included an explanation of the neutral position of the spine and also the correct breathing techniques used during Pilates. After the introductory session the eight-week mat Pilates exercise program commenced. The program consisted of three non-consecutive sessions a week, 60 minutes in duration, for eight-weeks with increasing intensity. All sessions started with breathing, followed by a flowing system from standing, to sitting, to lying down exercises and ended with the rest position. 

Statistical analysis consisted of basic statistics to determine pre- and post-test means and standard deviations. A paired samples t-test was used to determine if a significant change took place in the measurements at post-test. Differences in measurements were compared using a one-way analysis of variance (ANOVA) using a Dunnett T3 *post-hoc* analysis. Data was analyzed using commercial software (Statistical Package for Social Sciences (SPSS) Version 17, Chicago, IL) and statistical significance set at p ≤ 0.05.

## RESULTS

At pre-test, the CG and IG were heterogeneous for resting HR (p = 0.047). At pre-test, the groups were, however, homogenous for resting systolic BP (p = 0.736), resting diastolic BP (p = 0.143), fasting blood glucose levels (p = 0.319), blood TC (p = 0.754) and blood TG (p = 0.474). Following the eight-week mat Pilates program, the IG only demonstrated a significant (p ≤ 0.05) decrease in systolic BP (p = 0.040) from 135.84 ± 14.66mmHg to 128.80 ± 16.36mmHg and a significant increase in blood glucose (p = 0.000) from 5.07 ± 0.46mmol.L^-1^ to 5.83 ± 0.57mmol.L^-1^, whereas resting HR (p = 0.148) (from 68.80 ± 12.58beats.min^-1^ to 73.20 ± 11.46beats.min^-1^), resting diastolic BP (p = 0.342) (from 75.64 ± 10.10mmHg to 77.44 ± 9.32mmHg), blood TC (p = 0.073) (from 5.37 ± 0.99mmol.L^-1^ to 5.67 ± 1.04mmol.L^-1^) and blood TG (p = 0.384) (from 1.77 ± 0.88mmol.L^-1^ to 1.92 ± 0.87mmol.L^-1^) did not produce any significant changes following the eight-week exercise programme. Despite the CG demonstrating no significant difference in resting systolic BP (p = 0.705), resting diastolic BP (p = 0.471), blood TC (p = 0.054), blood TG (p = 0.321), the CG did demonstrate significant changes in resting HR and blood glucose from pre- to post-test (p = 0.000 and p = 0.001, respectively) (Table-II).

## DISCUSSION

As individuals age, physiological functioning decreases and most of the elderly develop at least one of the following chronic diseases: hypertension (49%), arthritis (36%), heart disease (31%), cancer (20%) and/or diabetes (15%).^20^ It was however, stated that exercise has major benefits for the cardiovascular system (CVD, hypertension, stroke, arrhythmias and peripheral artery disease) and metabolism (body fat, glucose homeostasis and insulin levels and lipids).^21^ Results obtained from the present study, however, indicate that eight-weeks of mat Pilates did not produce improvements in any of the cardiometabolic variables tested except systolic BP, which is a desirable finding as hypertension is one of the most significant risk factors for cerebrovascular and CVD.^22^

The present study correlates with the findings of Jago et al.^14^ regarding diastolic BP by indicating that no significant changes occurred in diastolic BP at post-test. In this regard, Jago et al*.*^14^ found non-significant changes in systolic and diastolic BP (from 108.2±9.4 mmHg to 102.5±5.6 mmHg and from 62.7±6.2 mmHg to 58.9±6.9 mmHg, respectively), following four-weeks of Pilates training in girls. Even though no significant change occurred in diastolic blood pressure (BP), the present study demonstrated a significant reduction in systolic BP. Brandaorondon et al.^23^ found that exercise significantly decreases BP in hypertensive elderly and provokes a decrease in BP that lasts for 22 hours, the moderately hypertensive pre-test systolic BP value should not be neglected. This reduction found in BP after exercise in elderly hypertensive individuals can be associated with a decrease in stroke volume and left ventricular end-diastolic volume.^23^ The reduction in BP can also be attributed to a diet high in cholesterol or fat that might result in fatty deposits called plaques to clot the arteries, thus resulting in narrowing of the arteries and increasing pressure. However, this cannot be confirmed in the present study since no dietary analyses were conducted. Exercise also exerts beneficial effects on blood lipids, but since the separate measures of high-density lipoprotein cholesterol (HDL-C) and low-density lipoprotein cholesterol (LDL-C) were not assessed, the vascular remodeling of existing arteries can also not be confirmed in the present study. In general, obesity and overweight are considered controllable risk factors for hypertension,^18^ but with a more thorough investigation^24^ found that body mass index (BMI), WHR, and skinfolds adiposity have some independent effect on the risk of elevated BP, where waist and hip circumferences are most important variables in women for obesity and BP. As such, it can be assumed that the decrease found in BP after completion of the eight-week mat Pilates programme in the present study can be as a result of the decrease found in waist circumference (WC) from 83.50 ± 12.79mm to 81.44 ± 9.90mm at post-test. Another influential mechanism in the decrease in systolic BP could have been due to total body relaxation and mental calmness as a result of correct breathing during the mat Pilates intervention,^16^ as mental or emotional stress activates nervous and endocrine responses, such as an increase in epinephrine and norepinephrine stimulation that results in an increase in BP, while preparing the body for physical activity.^2^^,^^25^

Skeletal muscle is the primary site of glucose disposal, therefore by increasing skeletal mass might be an effective way to improve insulin action.^26^ Based on this statement, a decrease in blood glucose in the IG would have been expected as the present study demonstrated a significant (p = 0.006) increase in lean body mass (LBM) (from 46.67 ± 6.33kg to 48.04 ± 7.52kg) in the IG. However, this was not the case, instead both the control group (CG) and the IG demonstrated a significant increase in blood glucose. The mean fasting glucose levels are significantly higher during the colder than warmer months.^27^ The present study was conducted during the transition from a warmer to a colder season, hence the possibility of the increase in blood glucose observed in the present study. The significant increase in glucose from pre- to post-test can also possibly be due to different instead of fixed nutritional consumption followed by each subject, several days prior to testing.

The present study found no significant changes in TC and TG after completion of an eight-week mat Pilates programme. The results found on blood lipids in the present study was not attributable to any of the anthropometric variables as the majority of TG are stored in adipose cells^28^ and favourable changes from pre- to post-test was observed in body fat percentage (BF%) (from 33.85 ± 6.67% to 32.23 ± 5.82%), WC (from 83.50 ± 12.79 millimeters to 81.44 ± 9.90 millimeters), fat mass (FM) (from 25.03 ± 9.53 kilograms to 23.69 ± 8.06 kilograms) and LBM (from 46.67 ± 6.33 kilograms to 48.04 ± 7.52 kilograms) (data not shown). A possible reason might have been due to an increase in HDL-C without a corresponding decrease in LDL-C, but as the CG also showed an increase in TC, this could not be a reasonable assumption. Uncontrollable factors affecting lipids are genetics, age and gender,^29^ but as only elderly women were used in the study age and gender could not have influenced the outcome. Boardley et al,^29^ however, indicated that dietary intake also influences blood lipids. As such, the observation might also be, as in the case of glucose, attributed to different instead of fixed nutritional consumption followed by each subject several days prior to testing. However, again this cannot be confirmed since no dietary analyses were conducted.

## CONCLUSIONS

The present study demonstrated that eight-weeks of Pilates is sufficient to produce a significant improvement in systolic BP, however, due to the contradictory nature of the other cardiometabolic variables with the findings of previous studies, it is difficult to establish a case for using Pilates as a substitute for more conventional forms of exercising when exclusively attempting to favourably alter cardiometabolic parameters in elderly women. 
